# A phase 1, open-label study of LCAR-B38M, a chimeric antigen receptor T cell therapy directed against B cell maturation antigen, in patients with relapsed or refractory multiple myeloma

**DOI:** 10.1186/s13045-018-0681-6

**Published:** 2018-12-20

**Authors:** Wan-Hong Zhao, Jie Liu, Bai-Yan Wang, Yin-Xia Chen, Xing-Mei Cao, Yun Yang, Yi-Lin Zhang, Fang-Xia Wang, Peng-Yu Zhang, Bo Lei, Liu-Fang Gu, Jian-Li Wang, Nan Yang, Ru Zhang, Hui Zhang, Ying Shen, Ju Bai, Yan Xu, Xu-Geng Wang, Rui-Li Zhang, Li-Li Wei, Zong-Fang Li, Zhen-Zhen Li, Yan Geng, Qian He, Qiu-Chuan Zhuang, Xiao-Hu Fan, Ai-Li He, Wang-Gang Zhang

**Affiliations:** 1 Department of Hematology, The Second Affiliated Hospital of Xi’an Jiaotong University, 157 West 5th Road, Xi’an, 710004 ShaanXi China; 2 National-Local Joint Engineering Research Center of Biodiagnostics & Biotherapy, The Second Affiliated Hospital of Xi’an Jiaotong University, Xi’an, 710004 ShaanXi China; 3 Department of Clinical Laboratory, The Second Affiliated Hospital of Xi’an Jiaotong University, Xi’an, 710004 ShaanXi China; 4 Nanjing Legend Biotech Inc., Nanjing, 210000 Jiangsu China

**Keywords:** Chimeric antigen receptor, CAR T, BCMA, Multiple myeloma, Relapsed, Refractory

## Abstract

**Background:**

Chimeric antigen receptor (CAR) T cell therapy has demonstrated proven efficacy in some hematologic cancers. We evaluated the safety and efficacy of LCAR-B38M, a dual epitope-binding CAR T cell therapy directed against 2 distinct B cell maturation antigen epitopes, in patients with relapsed/refractory (R/R) multiple myeloma (MM).

**Methods:**

This ongoing phase 1, single-arm, open-label, multicenter study enrolled patients (18 to 80 years) with R/R MM. Lymphodepletion was performed using cyclophosphamide 300 mg/m^2^. LCAR-B38M CAR T cells (median CAR+ T cells, 0.5 × 10^6^ cells/kg [range, 0.07 to 2.1 × 10^6^]) were infused in 3 separate infusions. The primary objective is to evaluate the safety of LCAR-B38M CAR T cells; the secondary objective is to evaluate the antimyeloma response of the treatment based on the general guidelines of the International Myeloma Working Group.

**Results:**

At data cutoff, 57 patients had received LCAR-B38M CAR T cells. All patients experienced ≥ 1 adverse events (AEs). Grade ≥ 3 AEs were reported in 37/57 patients (65%); most common were leukopenia (17/57; 30%), thrombocytopenia (13/57; 23%), and aspartate aminotransferase increased (12/57; 21%). Cytokine release syndrome occurred in 51/57 patients (90%); 4/57 (7%) had grade ≥ 3 cases. One patient reported neurotoxicity of grade 1 aphasia, agitation, and seizure-like activity. The overall response rate was 88% (95% confidence interval [CI], 76 to 95); 39/57 patients (68%) achieved a complete response, 3/57 (5%) achieved a very good partial response, and 8/57 (14%) achieved a partial response. Minimal residual disease was negative for 36/57 (63%) patients. The median time to response was 1 month (range, 0.4 to 3.5). At a median follow-up of 8 months, median progression-free survival was 15 months (95% CI, 11 to not estimable). Median overall survival for all patients was not reached.

**Conclusions:**

LCAR-B38M CAR T cell therapy displayed a manageable safety profile and demonstrated deep and durable responses in patients with R/R MM.

**Trial registration:**

ClinicalTrials.gov, NCT03090659; Registered on March 27, 2017, retrospectively registered

**Electronic supplementary material:**

The online version of this article (10.1186/s13045-018-0681-6) contains supplementary material, which is available to authorized users.

## Background

Multiple myeloma is a neoplasm of plasma cells that represents 13% of hematologic cancers and 1% of all cancers [1]. Despite the development of novel agents such as proteasome inhibitors, immunomodulatory drugs, and monoclonal antibodies that have significantly improved patient outcomes [2–10], multiple myeloma is still considered to be an incurable disease. The majority of patients will eventually relapse or become refractory to treatment; therefore, the development of new therapies for multiple myeloma remains a major unmet medical need [3].

B cell maturation antigen (BCMA) is a member of the tumor necrosis factor superfamily and represents an ideal target for multiple myeloma immunotherapy. BCMA is widely expressed on the surface of multiple myeloma cells, but has low expression on normal cells and no expression on CD34^+^ hematopoietic cells [11–14].

Chimeric antigen receptor (CAR) T cell therapy has emerged as an efficacious treatment in some relapsed/refractory hematologic cancers. CAR T cell therapies have been approved in the US for the treatment of relapsed or refractory acute lymphoblastic leukemia and large B cell lymphoma based on the high level of durable remissions that were achieved [15–19]. In multiple myeloma, 2 phase 1 studies using CAR T cells targeting BCMA in heavily pretreated patients showed encouraging preliminary responses [20, 21]. In the CAR-BCMA study, of the 16/24 patients treated at the highest CAR T cell dose, 13 patients achieved a partial response (PR) or better, demonstrating an overall response rate (ORR) of 81% [20]. In updated results of the bb-2121 study (*N* = 43), an ORR of 95.5% was observed in the 22 evaluable patients treated with the highest CAR T cell dose range [21]. The promising efficacy shown in these preliminary studies suggests that BCMA-targeted CAR T cell therapy may be a viable approach in the treatment of relapsed or refractory multiple myeloma.

LCAR-B38M is a dual epitope-binding CAR T cell therapy directed against 2 distinct BCMA epitopes. The bi-epitope BCMA-binding moieties confer high avidity binding and distinguish LCAR-B38M from other BCMA CAR constructs. Here, we report results from the full data set of 57 patients from our study site’s clinical experience with an ongoing phase 1 study of LCAR-B38M CAR T cell therapy in patients with relapsed or refractory multiple myeloma. Preliminary results of the first 35 patients treated at our site were presented at scientific meetings in 2017 [22].

## Methods

### Study design

A phase 1 trial of LCAR-B38M CAR T cells (LEGEND-2) has been explored at multiple centers in China, each with their own protocol for lymphodepletion and timing of CAR T cell administration. Study enrollment has ended at all 4 of these sites.

The analysis reported here represents the clinical experience (*N* = 57) from The Second Affiliated Hospital of Xi’an Jiaotong University, which used cyclophosphamide alone as the lymphodepleting therapy and infused CAR T cells in 3 split doses. The study was initiated on March 30, 2016, and the analysis reported here is from a data cutoff date of February 6, 2018. A study schema is presented in Additional file 1.

Eligible patients were between 18 and 80 years of age with confirmed diagnosis of relapsed or refractory multiple myeloma. Multiple myeloma was defined by the International Myeloma Working Group (IMWG) criteria [23], and relapse was defined by the National Comprehensive Cancer Network criteria [24].

All patients underwent leukapheresis to obtain peripheral blood mononuclear cells from which T cells were purified. CD3^+^ T cells were transduced with LCAR-B38M lentiviral vector to express anti-BCMA CAR (Additional file 2). The engineered T cells were further expanded ex vivo under interleukin-2 stimulation. Lymphodepletion using 3 doses of cyclophosphamide 300 mg/m^2^ on days − 5, − 4, and − 3 was followed by infusion of the engineered LCAR-B38M CAR T cells 5 days after the initiation of lymphodepletion chemotherapy. The total weight-adjusted CAR+ T cell dose (median, 0.5 × 10^6^ cells/kg [range, 0.07 to 2.1 × 10^6^]) was split into 3 infusions (20, 30, and 50% of total dose) administered over 7 days.

### Objectives and assessments

The primary objective is to evaluate the safety of LCAR-B38M, and the secondary objective is to assess the antimyeloma activity of CAR T cell therapy. Adverse events (AEs) were identified and toxicity was graded using the National Cancer Institute Common Terminology Criteria for Adverse Events, v.4.03. Cytokine release syndrome (CRS) was assessed using the modified criteria proposed by Lee et al. [25]. No prophylactic treatment was given for CRS.

Response assessments were based on the general guidelines from the IMWG [26, 27]. Disease evaluations were conducted using serum quantitative immunoglobulin or free light chain levels followed by immunofixation (serum and urine) and bone marrow biopsy/flow cytometry after normalization of serum quantitative immunoglobulin and free light chain levels. Two eight-color flow cytometry panels were used for minimal residual disease (MRD) assessment. The first panel included CD38, CD45, CD19, CD56, CD27, CD81, CD200, and CD20 antibodies. The second panel included CD38, CD138, CD19, CD45, CD117, and CD28 antibodies; cytoplasmic kappa and cytoplasmic lambda antibodies were used to assess clonality. Samples were analyzed on a Canto II flow cytometer (Becton Dickenson). The sensitivity of MRD assessment was up to 1 × 10^−4^.

BCMA expression was measured in malignant plasma cells from either bone marrow or plasmacytoma by flow cytometry. Whole blood was collected at serial time points to measure the number of LCAR-B38M CAR T cells using an exploratory quantitative polymerase chain reaction (PCR) assay. Preliminary assay results were generated with a research grade real-time PCR assay, which represents a semi-quantitative assessment of CAR gene copy numbers over time.

### Statistical analysis

The all-treated population included all patients who received at least 1 dose of LCAR-B38M CAR T cells and was used for all safety and efficacy analyses. Categorical variables were summarized using frequency counts and percentages. Continuous variables included the mean, standard deviation, median, and range (minimum and maximum) and were summarized using descriptive statistics.

The ORR was defined as the proportion of patients who achieved a complete response (CR), very good partial response (VGPR), or PR, after receiving study treatment. Two-sided 95% exact confidence intervals (CIs) based on exact method of binomial distribution were calculated for each response category. The median duration of response, progression-free survival, and overall survival and corresponding 95% CI were calculated using Kaplan-Meier method.

## Results

### Patients

At data cutoff (February 6, 2018), 57 patients were enrolled in the study site and had received LCAR-B38M CAR T cell therapy. The median age was 54 years (range, 27 to 72), and more men (60%) were enrolled in the study (Table 1). The median number of prior lines of therapy was 3 (range, 1 to 9), including prior proteasome inhibitor therapy (68%), immunomodulatory agents (86%), and both proteasome inhibitors and immunomodulatory agents (60%; Table 1). At the time of enrollment, 37% of patients had stage III disease as assessed by the International Staging System. The median duration of follow-up for all patients was 8 months (range, 0.7 to 20.7). Due to clinical practice differences, refractoriness could not be systematically assessed by the study physician.

**Table 1:** Demographics and baseline disease characteristics

	Total (*N* = 57)
Age, *n* (%)	
< 65	46 (81)
≥ 65	11 (19)
Mean (SD)	55 (9)
Median (range)	54 (27 to 72)
Sex, *n* (%)	
Male	34 (60)
Female	23 (40)
ECOG performance status, *n* (%)	
0	21 (37)
1	27 (47)
2	9 (16)
ISS stage, *n* (%)	
I	15 (26)
II	14 (25)
III	21 (37)
Unknown	7 (12)
Type of myeloma^a^, *n* (%)	
IgG	25 (44)
IgA	15 (26)
Light chain	17 (30)
Kappa	7 (12)
Lambda	10 (18)
Time from initial MM diagnosis, years	
Mean (SD)	4 (2)
Median (range)	4 (1 to 9)
Number of prior lines of therapy, n	
Mean (SD)	3 (2)
Median (range)	3 (1 to 9)
Autologous stem cell transplantation, *n* (%)	10 (18)
Prior therapies, *n* (%)	
Proteasome inhibitors	39 (68)
Bortezomib	39 (68)
Carfilzomib	1 (2)
Immunomodulatory agents	49 (86)
Lenalidomide	25 (44)
Pomalidomide	2 (4)
Thalidomide	39 (68)
Proteasome inhibitors + immunomodulatory agents	34 (60)

*ECOG* Eastern Cooperative Oncology Group, *IgA* immunoglobulin A, *IgG* immunoglobulin G, *ISS* International Staging System, *SD* standard deviation

^a^The type of myeloma was assessed per International Myeloma Working Group criteria for diagnosis of multiple myeloma [23]

### Safety

AEs were reported in all patients. The most common (≥ 40%) AEs of any grade were pyrexia (91%), CRS (90%), thrombocytopenia (49%), and leukopenia (47%; Table 2). Grade ≥ 3 AEs were reported in 37 patients (65%); the most common (≥ 20%) grade ≥ 3 events were leukopenia (30%), thrombocytopenia (23%), and aspartate aminotransferase increased (21%).

**Table 2:** Adverse events that occurred in at least 10% of patients

AE, *n* (%)	All grade	Grades 1–2	Grade ≥ 3
Pyrexia	52 (91)	41 (72)	11 (19)
Cytokine release syndrome^a^	51 (90)	47 (83)	4 (7)
Thrombocytopenia	28 (49)	15 (26)	13 (23)
Leukopenia	27 (47)	10 (18)	17 (30)
AST increased	22 (39)	10 (18)	12 (21)
Anemia	17 (30)	7 (12)	10 (18)
Hypotension	12 (21)	9 (16)	3 (5)
ALT increased	10 (18)	10 (18)	0
Cough	10 (18)	10 (18)	0
Disseminated intravascular coagulation	10 (18)	9 (16)	1 (2)
Hypocalcemia	9 (16)	7 (12)	2 (4)
Hyponatremia	8 (14)	5 (9)	3 (5)
Dyspnea	6 (11)	6 (11)	0
Nausea	6 (11)	6 (11)	0

*ALT* alanine aminotransferase, *AST* aspartate aminotransferase

^a^Cytokine release syndrome assessed using modified criteria by Lee et al. [25]

CRS, assessed according to Lee et al. criteria [25], was mostly grades 1 (47%) and 2 (35%); 4 patients (7%) had grade 3 cases (Table 2). Liver function abnormalities, particularly aspartate aminotransferase increased, were the most common signs of end organ injury among patients with CRS (Table 3). Coagulopathies were also observed in patients who had CRS. Among patients who had CRS and available laboratory assessments, prolongation of activated partial thromboplastin time and decreased fibrinogen occurred in 67 and 37% of patients, respectively. Kidney, pulmonary, and vascular system abnormalities were less frequent (2 to 35%) than those observed for the liver (Table 3).

**Table 3:** End organ abnormalities among patients with cytokine release syndrome

	Total patients with CRS (*N* = 51)		
	All grades	Grades 1–2	Grades 3–4
Liver			
ALT high	25 (49)	24 (47)	1 (2)
AST high	40 (78)	23 (45)	17 (33)
Bilirubin high	22 (43)	22 (43)	0
Kidney			
Creatinine high	18 (35)	15 (29)	3 (6)
Pulmonary and vascular systems			
Hypotension	12 (24)	9 (18)	3 (6)
Hypoxia	4 (8)	4 (8)	0
Respiratory failure	1 (2)	0	1 (2)
Coagulopathy^a^			
aPTT prolonged	31 (67)	28 (61)	3 (7)
Fibrinogen decreased	17 (37)	0	17 (37)

*ALT* alanine aminotransferase, *aPTT* activated partial thromboplastin time, *AST* aspartate aminotransferase, *CRS* cytokine release syndrome

^a^Only 46 patients had laboratory test results available (*N* = 46 was used as the denominator)

The median time to onset of any grade CRS was 9 days (range, 1 to 19). Tocilizumab (46%), vasopressor (11%), and supplemental oxygen (35%) were used to manage the CRS. One patient, who had received a dose of 1.8 × 10^6^ CAR+ T cells/kg, developed grade 2 CRS. While the CRS was resolving, the patient developed sudden onset of respiratory distress 22 days after receiving the first dose of CAR T cells. The patient required intubation for ventilator support and died 1 h later with cause of death reported as grade 5 pulmonary embolism and acute coronary syndrome. The intubation was performed as part of final resuscitation efforts and not specifically to treat the CRS. Except for the patient who had grade 5 AEs, all CRS events resolved, with a median duration of 9 days (range, 3 to 57).

Neurotoxicity was observed in 1 patient, dosed at 1.0 × 10^6^ CAR+ T cells/kg, who developed grade 1 aphasia, agitation, and seizure-like activity. The patient was treated with 10 mg diazepam, and the events resolved within 1 day.

### Efficacy

**Fig. 1:**
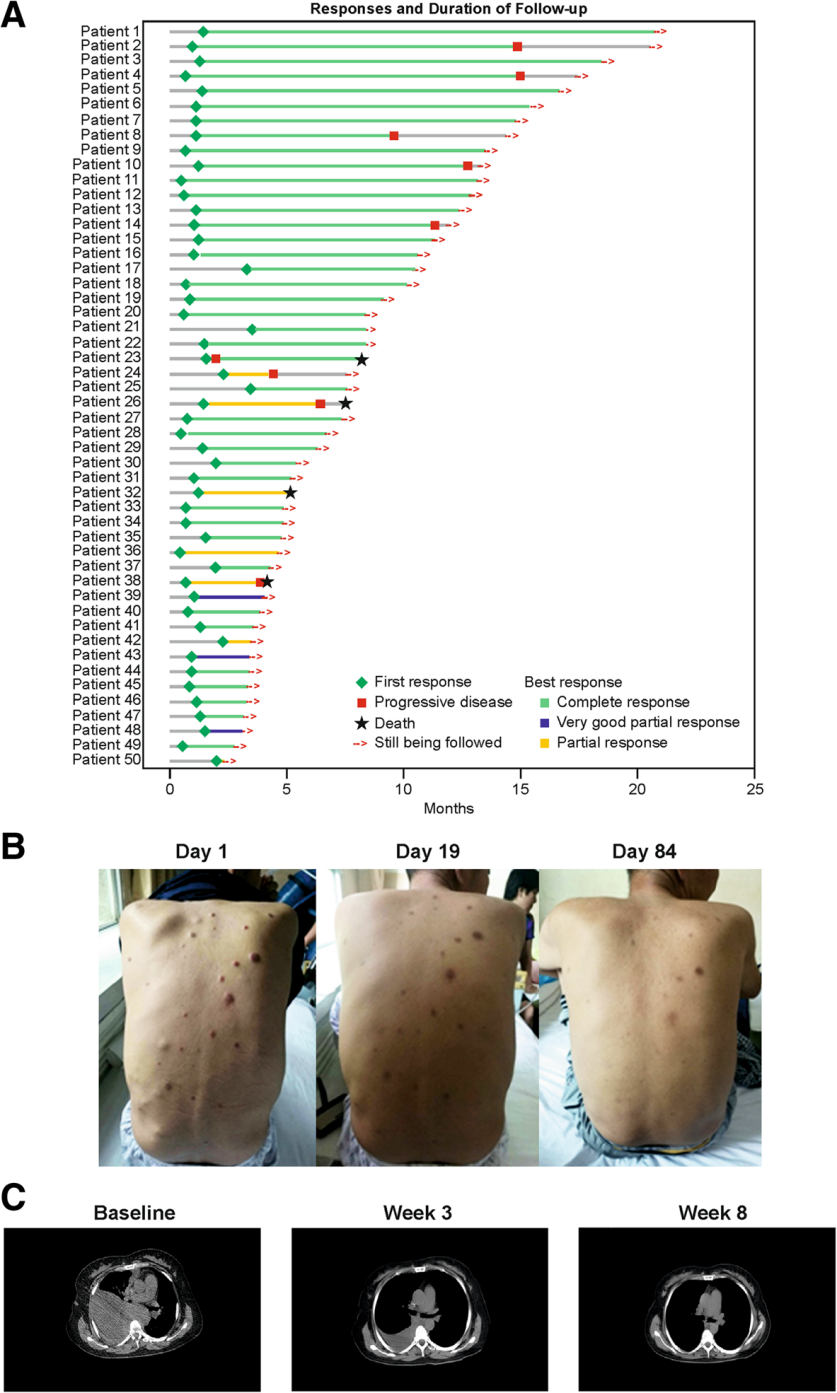
Efficacy assessments of LCAR-B38M. **a** Individual patient response and duration of follow-up for patients who achieved at least a PR (*n* = 50). **b** One patient manifested extramedullary myeloma and wide subcutaneous metastasis. Examination on day 1, day 19, and day 84 after LCAR-B38M CAR T cell infusion showed regression of the subcutaneous metastases. **c** CT scans of a patient with an extramedullary lesion beside the thoracic vertebrae and severe pleural effusion at baseline and at week 3 and week 8 following LCAR-B38M CAR T cell infusion. It should be noted that the patient received thoracentesis intermittently before the CAR T treatment but did not receive thoracentesis or intrathoracic injection of drugs after treatment

In total, 50 of 57 patients responded; the ORR was 88% (95% CI, 76 to 95). CR was achieved by 39 patients (68%; 95% CI, 55 to 80), VGPR was achieved by 3 patients (5%; 95% CI, 1 to 15), and PR was achieved by 8 patients (14%; 95% CI, 6 to 26; Fig. 1a). MRD was negative for 36/57 patients (63%; 95% CI, 49 to 76), as assessed by bone marrow eight-color flow cytometry assay, and all MRD negative patients achieved CR. The median time to initial response was 1 month (range, 0.4 to 3.5).

LCAR-B38M CAR T cells also demonstrated activity in patients with extramedullary disease, with observed decreases in tumor mass (Fig. 1b, c).

**Fig. 2:**
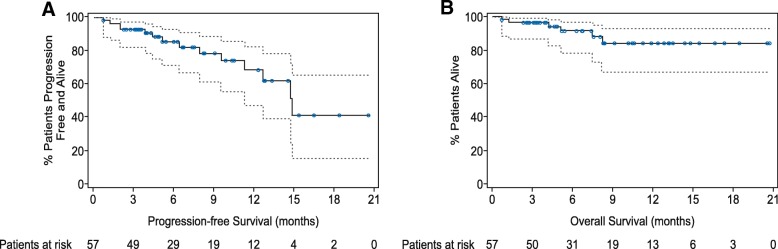
Progression-free survival and overall survival. **a** The Kaplan-Meier curve for progression-free survival for all treated patients. The median progression-free survival was 15 months (95% CI, 11 to not estimable). **b** The Kaplan-Meier curve for overall survival for all treated patients. The median overall survival was not reached. The dotted lines represent 95% confidence intervals

At data cutoff, 10 patients (20%) who achieved a PR or better subsequently progressed. The Kaplan-Meier estimate of median duration of response was 14 months (95% CI, 12 to not estimable). The median progression-free survival was 15 months (95% CI, 11 to not estimable; Fig. 2a). Six patients died during the study due to disease progression (*n* = 4), suicide after disease progression (*n* = 1), and pulmonary embolism and acute coronary syndrome (*n* = 1). The median overall survival was not reached (Fig. 2b).

### BCMA expression and clinical response

Among the patients with BCMA expression data, 26/53 (49%) had < 40% BCMA expression and 27/53 (51%) had ≥ 40% BCMA expression (Additional file 3). BCMA expression did not appear to correlate with clinical response. An ORR of 92% was observed in patients with < 40% BCMA expression compared with an ORR of 82% in patients with ≥ 40% BCMA expression. Correlation between BCMA expression and median PFS or OS was not observed. Median PFS was 15 months for patients who had < 40% BCMA expression and 11 months for patients who had ≥ 40% BCMA expression. Median OS was not reached for both the < 40 and ≥ 40% BCMA expression groups (Additional file 3).

### Exposure and persistence of LCAR-B38M CAR T cells

The median number of total CAR+ T cells infused was 32.3 × 10^6^ (range, 3.3 to 126.2 × 10^6^). The body weight-adjusted median number of CAR+ T cells administered was 0.5 × 10^6^ cells/kg (range, 0.07 to 2.1 × 10^6^). The overall incidence and severity of CRS was higher with increasing median LCAR-B38M CAR T cell dose; however, the small number of patients with higher grade events limits the interpretation of the data. There was no clear relationship between LCAR-B38M CAR T cell dose and disease response (Additional file 4).

Among the patients who had blood samples for analysis (*n* = 31), the majority had peak levels of LCAR-B38M signal greater than 1 × 10^4^ copies/μg genomic DNA following infusion. LCAR-B38M CAR T cells in most patients (71%) were not detectable (signal below the lower limit of detection) in peripheral blood at 4 months, and only 5 patients showed CAR T cell persistence up to 10 months (Additional file 5).

### Biomarkers

Serum interleukin-6 increased transiently within the first month following LCAR-B38M CAR T cell infusion. The peak levels of interleukin-6 increased with severity of CRS (Additional file 6). Similar results were observed with interleukin-2, interleukin-8, interleukin-10, and tumor necrosis factor alpha.

Infusion of LCAR-B38M CAR T cells resulted in a rapid reduction of serum immunoglobulin levels in all patients. IgA and IgG levels eventually increased for some patients who also had disease progression (Additional file 7).

## Discussion

In this analysis of an ongoing phase 1, first-in-human clinical study, LCAR-B38M, a CAR T cell therapy directed against 2 distinct BCMA epitopes, displayed a manageable safety profile and demonstrated durable responses in patients with relapsed or refractory multiple myeloma.

CRS occurred in 90% of patients and were mostly grades 1 and 2 (83%), with 4 (7%) grade 3 cases. The overall response rate was 88%, and 39 patients (68%) achieved CR at a median follow-up of 8 months (range, 0.7 to 20.7).

All cases of CRS started with pyrexia with a median time to onset of 9 days (range, 1 to 19) after the first infusion of LCAR-B38M CAR T cells, suggesting that careful monitoring for fever during the first several days following CAR T cell infusion could be used in future studies to identify patients likely to require additional support for the CRS.

Cytopenias (thrombocytopenia and neutropenia) were common (54%); prolonged grade 4 cytopenias with duration exceeding 35 days occurred in 16% of patients. Infection AEs were reported, although the rate (9%) did not exceed that expected for patients with multiple myeloma [28].

Time to response was rapid, with a median time of 1 month (range, 0.4 to 3.5). Consistent efficacy was observed across all subgroups examined, including prior autologous stem cell transplant, age, number of prior therapies, and prior proteasome and immunomodulatory agent treatment. Ten responders (20%) progressed or died at data cutoff.

BCMA expression did not appear to correlate with clinical response and was highly variable, as has been described by others [20]. In the dose expansion phase of the bb-2121 study, BCMA expression is not an eligibility requirement, suggesting that clinical response might be independent of BCMA expression levels.

This was an exploratory study conducted to evaluate the effects of different preconditioning chemotherapy and treatment regimens for LCAR-B38M. Because this was the first clinical experience with LCAR-B38M, cyclophosphamide alone, rather than cyclophosphamide plus fludarabine, was used as the preconditioning treatment for safety considerations. Typically, CAR T cell therapy has a broad range of efficacious doses, and it is not uncommon for CAR T cell studies to start without a dose escalation phase. The dose used in our study was determined by both the sponsor and the investigator. Several factors were considered to determine the dose for each patient: the patient’s disease status, overall condition prior to infusion, and available total CAR T cells manufactured.

In this study, the median age of patients was 54 years, which is consistent with reported differences in the median age at diagnosis between Chinese and Western patients with multiple myeloma (59 vs. 70 years, respectively) [1, 29, 30]. Taking the median age of patients into consideration, a median of 3 prior lines of therapy and ISS stage III disease in 37% of patients suggest that the patients in this study had advanced myeloma.

The 88% ORR observed in this study was higher than that observed with CAR-BCMA [20, 31]. Among the 10 patients treated at the lowest dose of 0.3 × 10^6^ CAR-BCMA CAR+ T cells/kg, only 2 patients (20.0%) achieved PR or better. For the 16 patients treated at the highest dose of 9 × 10^6^ CAR-BCMA CAR+ T cells/kg, 13 (81%) achieved PR or better [20, 31]. The observed difference in response may be related to the design of the CAR and the dose of CAR+ T cells infused. The CAR-BCMA design incorporates a 11D-5-3 single anti-BCMA single chain variable region, a CD28 costimulatory domain, and a CD3ζ T cell activation domain [20, 31] and is different from the design of LCAR-B38M, which binds to 2 distinct BCMA epitopes. Although it is not possible to compare across studies, the efficacious doses used in the CAR-BCMA study were also considerably higher than those of LCAR-B38M used in this study (median dose of 0.5 × 10^6^ CAR+ T cells/kg [range, 0.07 to 2.10 × 10^6^]).

The bb-2121 study (*N* = 43) tested a range of study doses from 50 × 10^6^ to 800 × 10^6^ CAR+ T cells. Among the 22 evaluable patients in the cohort receiving > 150 × 10^6^ CAR+ T cells, the ORR was 95.5% [21]. The final doses under evaluation for the phase 2 study of bb-2121 are 150 to 450 × 10^6^ CAR+ T cells, which are higher than the doses used in this study with LCAR-B38M (median total CAR+ T cells = 32.3 × 10^6^). The dual epitope-binding of LCAR-B38M, which confers high avidity binding, may result in clinical responses at lower cell doses, with less toxicity.

## Conclusions

This ongoing first-in-human study has provided initial proof-of-concept that dual epitope-binding LCAR-B38M CAR T cells may be a highly effective therapy for patients with relapsed or refractory multiple myeloma. LCAR-B38M displayed a manageable safety profile consistent with its known mechanism of action. Further clinical evaluation of this promising and potentially transformational treatment approach is underway, with an ongoing phase 1b/2 study in the US (ClinicalTrials.gov, NCT03548207).

## Additional files

Additional file 1: Study Schema. Figure showing the study schema. (DOCX 66 kb)

Additional file 2: LCAR-B38M lentiviral construct. Figure depicting the LCAR-B38M lentiviral construct. (DOCX 17 kb)

Additional file 3: Relationship between BCMA expression and clinical response, progression-free survival, and overall survival. Overall response rate, progression-free survival, and overall survival by BCMA expression. (DOCX 15 kb)

Additional file 4: Relationship between dose and response. Relationship between CAR+ cell dose and complete response. (DOCX 80 kb)

Additional file 5: Genomic copies of LCAR-B38M. Copies of LCAR-B38M per microgram of genomic DNA. (DOCX 90 kb)

Additional file 6: Interleukin-6 levels by cytokine release syndrome grade. Peak IL-6 levels by CRS grade. (DOCX 47 kb)

Additional file 7: Immunoglobulin levels following LCAR-B38M infusion. Levels of serum IgA, IgG, lambda chain, and kappa chain following CAR T cell infusion. (DOCX 281 kb)

## Abbreviations

AE: Adverse event
ALT: Alanine aminotransferase
aPTT: Activated partial thromboplastin time
AST: Aspartate aminotransferase
BCMA: B cell maturation antigen
CAR: Chimeric antigen receptor
CI: Confidence interval
CR: Complete response
CRS: Cytokine release syndrome
ECOG: Eastern Cooperative Oncology Group
IgA: Immunoglobulin A
IgG: Immunoglobulin G
IMWG: International Myeloma Working Group
ISS: International Staging System
MM: Multiple myeloma
MRD: Minimal residual disease
ORR: Overall response rate
PCR: Polymerase chain reaction
PR: Partial response
R/R: Relapsed/refractory
SD: Standard deviation
VGPR: Very good partial response
