# Supplementary figures and images for: A phase 1, open-label study of LCAR-B38M, a chimeric antigen receptor T cell therapy directed against B cell maturation antigen, in patients with relapsed or refractory multiple myeloma
Source: J Hematol Oncol. 2018 Dec 20;11:141. doi: 10.1186/s13045-018-0681-6 (PMC6302465; doi:10.1186/s13045-018-0681-6)

**Additional File 1. Study Schema**

**
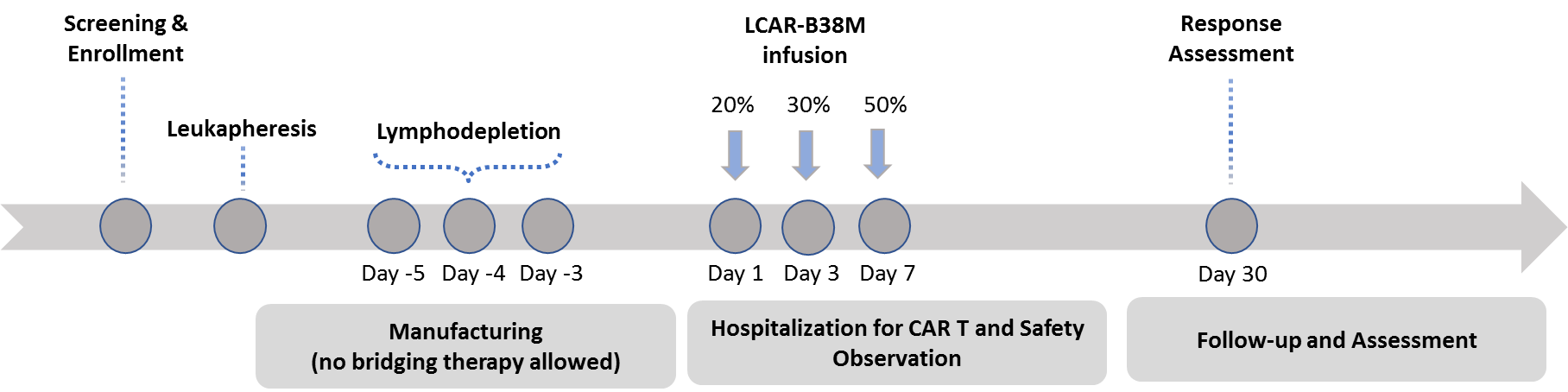
**

Supplement: Supplementary file 1 — Study Schema. Figure showing the study schema. (DOCX 66 kb) [file 13045_2018_681_MOESM1_ESM.docx]
