# Supplementary material for: A phase 1, open-label study of LCAR-B38M, a chimeric antigen receptor T cell therapy directed against B cell maturation antigen, in patients with relapsed or refractory multiple myeloma
Source: J Hematol Oncol. 2018 Dec 20;11:141. doi: 10.1186/s13045-018-0681-6 (PMC6302465; doi:10.1186/s13045-018-0681-6)
Supplement: Supplementary file 2 — LCAR-B38M lentiviral construct. Figure depicting the LCAR-B38M lentiviral construct. (DOCX 17 kb) [file 13045_2018_681_MOESM2_ESM.docx]

**Additional File 2. LCAR-B38M Lentiviral Construct**

**LCAR-B38M coding region**
