# Supplementary material for: A phase 1, open-label study of LCAR-B38M, a chimeric antigen receptor T cell therapy directed against B cell maturation antigen, in patients with relapsed or refractory multiple myeloma
Source: J Hematol Oncol. 2018 Dec 20;11:141. doi: 10.1186/s13045-018-0681-6 (PMC6302465; doi:10.1186/s13045-018-0681-6)
Supplement: Supplementary file 3 — Relationship between BCMA expression and clinical response, progression-free survival, and overall survival. Overall response rate, progression-free survival, and overall survival by BCMA expression. (DOCX 15 kb) [file 13045_2018_681_MOESM3_ESM.docx]

**Additional File 3. Relationship Between BCMA Expression and Clinical Response, Progression-Free Survival, and Overall Survival**

| **Efficacy, n (%)** | **BCMA <40%**  **n=26** | **BCMA ≥40%**  **n=27** |
| --- | --- | --- |
| Overall response rate (CR+VGPR+PR) | 24 (92) | 22 (82) |
| Complete response (CR) | 19 (73) | 17 (63) |
| Very good partial response (VGPR) | 1 (4) | 1 (4) |
| Partial response (PR) | 4 (15) | 4 (15) |
| **Median PFS (95%CI), month** | 15 (13­–NE) | 11 (6–NE) |
| **Median OS, month** | NR | NR |

BCMA=B-cell maturation antigen; CI=confidence interval; NE=not estimable; NR=not reached; OS=overall survival; PFS=progression-free survival. BCMA expression data available for 53 patients.
