# Supplementary material for: A phase 1, open-label study of LCAR-B38M, a chimeric antigen receptor T cell therapy directed against B cell maturation antigen, in patients with relapsed or refractory multiple myeloma
Source: J Hematol Oncol. 2018 Dec 20;11:141. doi: 10.1186/s13045-018-0681-6 (PMC6302465; doi:10.1186/s13045-018-0681-6)

**Additional File 4. Relationship Between Dose and Response.** No clear relationship between LCAR-B38M CAR T cell dose and disease response was observed. Patients who did or did not achieve a complete response (CR) both received a weight-adjusted median 0.5x10^6^ CAR+ T cells/kg.


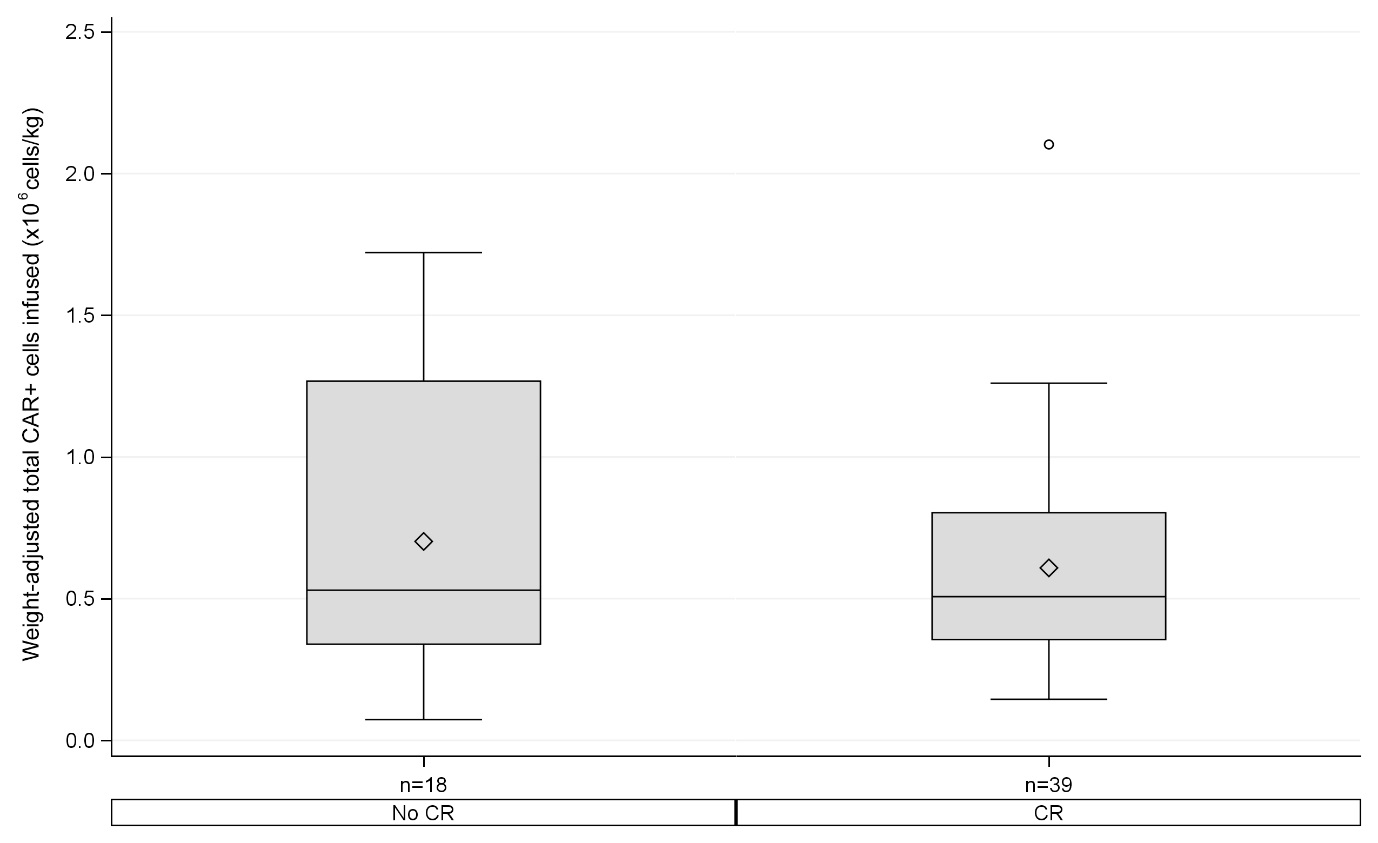

Supplement: Supplementary file 4 — Relationship between dose and response. Relationship between CAR+ cell dose and complete response. (DOCX 80 kb) [file 13045_2018_681_MOESM4_ESM.docx]
