# Supplementary material for: A phase 1, open-label study of LCAR-B38M, a chimeric antigen receptor T cell therapy directed against B cell maturation antigen, in patients with relapsed or refractory multiple myeloma
Source: J Hematol Oncol. 2018 Dec 20;11:141. doi: 10.1186/s13045-018-0681-6 (PMC6302465; doi:10.1186/s13045-018-0681-6)
Supplement: Supplementary file 5 — Genomic copies of LCAR-B38M. Copies of LCAR-B38M per microgram of genomic DNA. (DOCX 90 kb) [file 13045_2018_681_MOESM5_ESM.docx]

**Additional File 5. Genomic Copies of LCAR-B38M.** The presence of LCAR-B38M CAR T cells in peripheral blood was assessed by quantitative real-time polymerase chain reaction assay. The dotted line represents 1x10^3^ copies of BCMA CAR transgene per microgram of genomic DNA, which is the threshold for a detectable signal; values below this threshold are considered to be undetectable.
