# Supplementary material for: A phase 1, open-label study of LCAR-B38M, a chimeric antigen receptor T cell therapy directed against B cell maturation antigen, in patients with relapsed or refractory multiple myeloma
Source: J Hematol Oncol. 2018 Dec 20;11:141. doi: 10.1186/s13045-018-0681-6 (PMC6302465; doi:10.1186/s13045-018-0681-6)

**Additional File 6. Interleukin-6 Levels by Cytokine Release Syndrome Grade.** Peak levels of interleukin-6 increased with increasing severity of cytokine release syndrome.


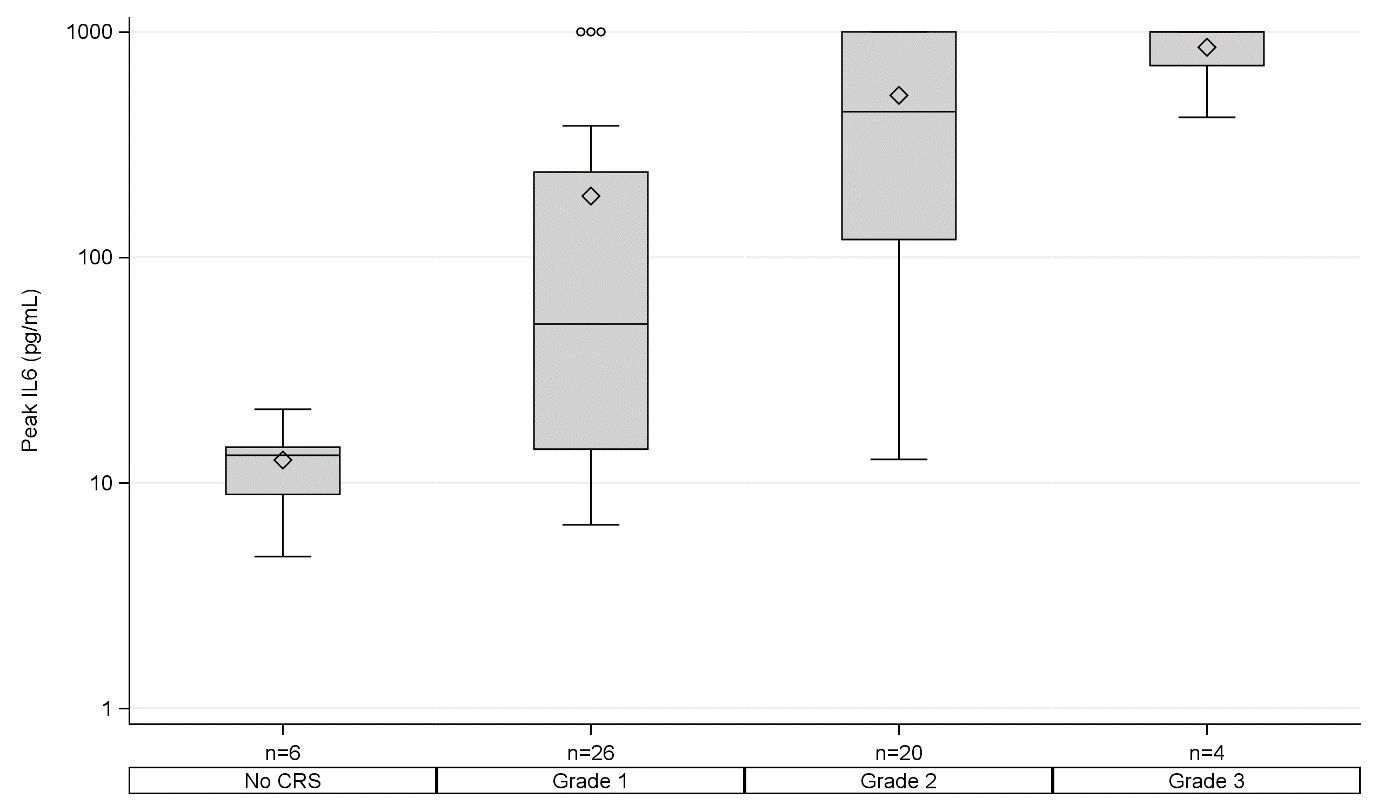

Supplement: Supplementary file 6 — Interleukin-6 levels by cytokine release syndrome grade. Peak IL-6 levels by CRS grade. (DOCX 47 kb) [file 13045_2018_681_MOESM6_ESM.docx]
