# Supplementary material for: A phase 1, open-label study of LCAR-B38M, a chimeric antigen receptor T cell therapy directed against B cell maturation antigen, in patients with relapsed or refractory multiple myeloma
Source: J Hematol Oncol. 2018 Dec 20;11:141. doi: 10.1186/s13045-018-0681-6 (PMC6302465; doi:10.1186/s13045-018-0681-6)

**Additional File 7.** **Immunoglobulin Levels Following LCAR-B38M Infusion.** Following LCAR-B38M infusion, immunoglobulin levels in all patients rapidly declined. The majority of patients who saw an increase in IgA and IgG levels after LCAR-B38M infusion had disease progression. Green lines represent patients who had disease progression. For serum lambda chain, the normal range is 8.3–27 mg/L; for serum kappa chain, the normal range is 6.7–22.4 mg/L.


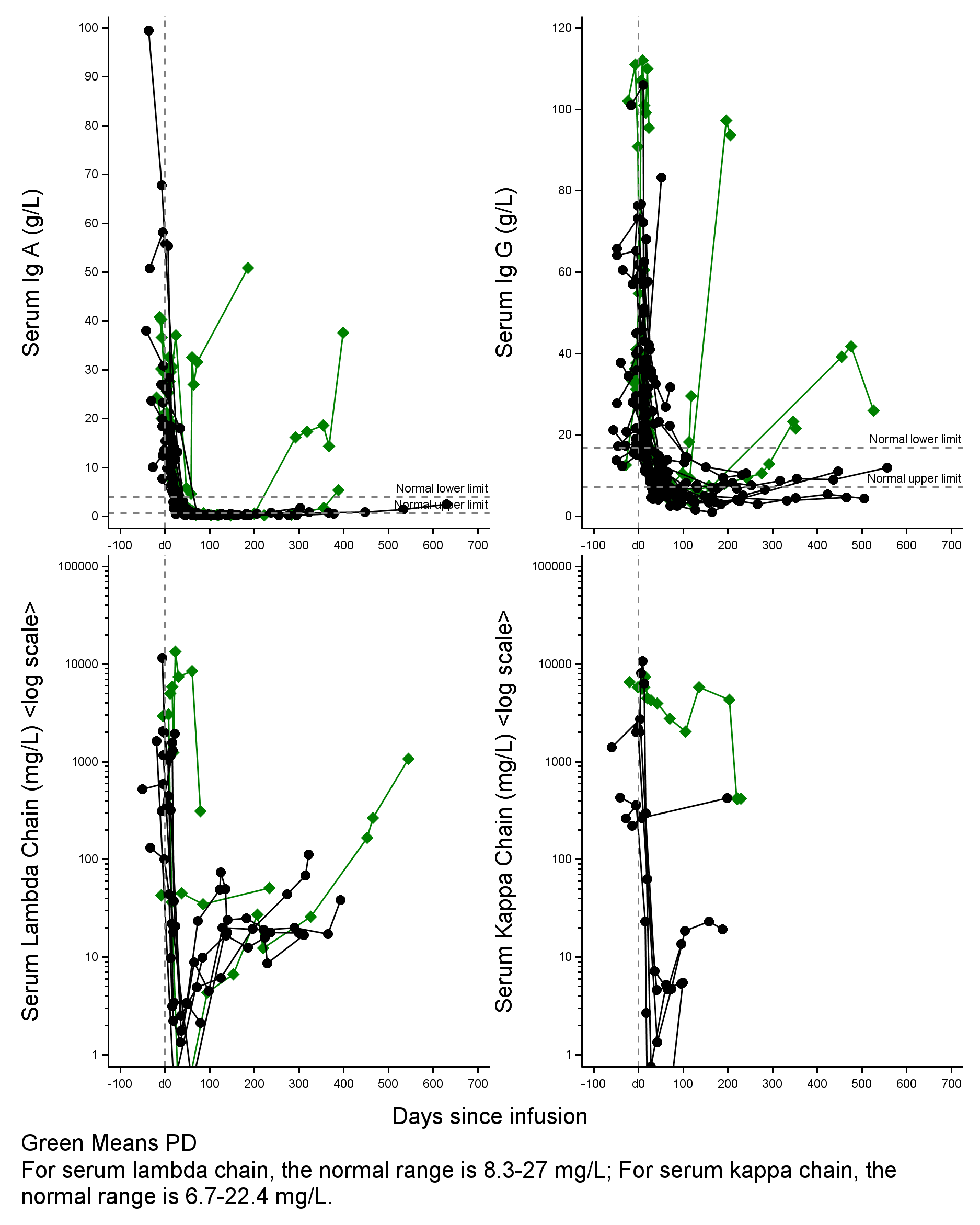

Supplement: Supplementary file 7 — Immunoglobulin levels following LCAR-B38M infusion. Levels of serum IgA, IgG, lambda chain, and kappa chain following CAR T cell infusion. (DOCX 281 kb) [file 13045_2018_681_MOESM7_ESM.docx]
